# Thermodynamic Analysis for the Refining Ability of Salt Flux for Aluminum Recycling

**DOI:** 10.3390/ma7085543

**Published:** 2014-07-30

**Authors:** Takehito Hiraki, Takahiro Miki, Kenichi Nakajima, Kazuyo Matsubae, Shinichiro Nakamura, Tetsuya Nagasaka

**Affiliations:** 1 Graduate School of Engineering, Tohoku University, Sendai 980-8578, Japan; E-Mails: miki@material.tohoku.ac.jp (T.M.); matsubae@m.tohoku.ac.jp (K.M.); t-nagasaka@m.tohoku.ac.jp (T.N.); 2 Center for Material Cycles and Waste Management Research, National Institute for Environmental Studies, Tsukuba 305-8506, Japan; E-Mail: nakajima.kenichi@nies.go.jp; 3 Graduate School of Economics, Waseda University, Tokyo 169-8050, Japan; E-Mail: nakashin@waseda.jp

**Keywords:** recycling of aluminum, thermodynamic analysis, impurity removal, salt flux, refining ability

## Abstract

The removability of impurities during the aluminum remelting process by oxidation was previously investigated by our research group. In the present work, alternative impurity removal with chlorination has been evaluated by thermodynamic analysis. For 43 different elements, equilibrium distribution ratios among metal, chloride flux and oxide slag phases in the aluminum remelting process were calculated by assuming the binary systems of aluminum and an impurity element. It was found that the removability of impurities isn’t significantly affected by process parameters such as chloride partial pressure, temperature and flux composition. It was shown that Ho, Dy, Li, La, Mg, Gd, Ce, Yb, Ca and Sr can be potentially eliminated into flux by chlorination from the remelted aluminum. Chlorination and oxidation are not effective to remove other impurities from the melting aluminum, due to the limited parameters which can be controlled during the remelting process. It follows that a proper management of aluminum scrap such as sorting based on the composition of the products is important for sustainable aluminum recycling.

## 1. Introduction

Total aluminum production was increased from 28 million tons in 1990 to 56 million tons in 2009. While the percentage of recycled aluminum in products has plateaued since 2000, 18 million tons of aluminum was recycled from scrap in 2009 [1]. Recycling aluminum is advantageous due to the large environmental and economic impact on raw materials and energy conservation. The aluminum remelting processes is essential for suppressing energy consumption since one metric ton of aluminum from bauxite requires about 17,000 kWh of electricity, while the same amount of recycled aluminum only consumes approximately 750 kWh [2]. According to [3], most aluminum is used in the form of alloys rather than pure metal with Cu, Fe, Mn, Mg, Si, and Zn mainly added to improve the chemical, physical and mechanical properties.

Nakajima *et al.* have analyzed the distribution tendency of alloying elements among gas, slag and metal phases in pyro metallurgical recycling processes of aluminum, steel, copper, lead, zinc, magnesium, and titanium [4,5,6,7,8] by evaluating the quantitative removal limits of impurities thermodynamically taking into account all relevant parameters such as the total pressure, the activity coefficient of the target impurity, the temperature, the oxygen partial pressure, and the activity coefficient of oxidation product. In regard to aluminum recycling, their results show that Be, Ca, and Mg can be removed by oxidation and Cd, Hg, and Zn can be eliminated by evaporation, but the removal of the other 39 elements, including Cu, Si, Fe, and Mn, is difficult since these have a strong tendency to remain in the metal phase. Many alloying elements are difficult to remove from end-of-life (EoL) aluminum products, including aluminum alloy scrap, because aluminum has a high ionization tendency and thermodynamic reactivity [9]. Accordingly, when the oxidation method is employed for the removal of impurities from aluminum scrap, aluminum tends to be preferentially oxidized into the slag. While aluminum is known as a well-recycled material in terms of quantity, in terms of quality, there is a risk of contamination by alloying elements in its recycling [10]. Hence, avoiding the contamination by alloying elements is crucial for sustainable recycling of aluminum alloys.

Salt flux treatment of aluminum scrap is a technological option to remove alloying elements or to extract aluminum from EoL aluminum products. Fluxes based on a mixture of molten salts are often utilized in the processing of molten aluminum. They are used in a passive role to protect the metal from oxidation and sometimes in an active role to remove the additive elements as impurities from molten aluminum. Fluxes are mainly blends of chloride and fluoride salts with additives to instill specific properties. Most fluxes are based on a mixture of NaCl and KCl [11,12,13]. Aluminum recovery during remelting process of aluminum scrap from turning process (known as swarf) [14] and Al composites [15] can be enhanced by use of NaCl–KCl based flux. Also, separation and recovery of aluminum alloys from aluminum dross was reported with utilization of BaCl_2_–NaCl–NaF flux [16]. Salt flux is useful and convenient for effective aluminum remelting. A limited number of reports are available concerning impurity removal from aluminum by salt flux such as distribution of Mg and Zn between KCl–NaCl and KCl–NaCl–AlCl_3_ melt and molten Al [17]. These reports suggest the possibility of impurity removal from molten aluminum alloy by salt flux treatment. However, to our knowledge, no quantitative exists with regard to the refining capacity of removing impurities using salt flux based remelting processes, despite the use of many alloying elements for enhancing the performance of products. This paper is aimed at clarifying the possibility of removing impurities by chlorination during the aluminum remelting process with salt flux. For 43 different elements that are likely to be contained in industrial aluminum alloys, the equilibrium distribution ratios among metal and chlorides in the aluminum remelting process were investigated, and the removal limits of impurities by chlorination was compared against those by oxidation.

## 2. Thermodynamic Methodology

The driving force of a chlorination reaction can be determined by the Gibbs free energy of the intended chemical reaction. Furthermore, if an element is distributed by reaction with chlorine gas into different phases such as molten metal and chloride, the distribution of the element can be quantitatively evaluated by calculating the equilibrium constant from the change in the Gibbs free energy and then converting it into its concentration in each of the phases. For such conditions, the following relationships were used to obtain the parameters controlling the distribution of elements among the metal and chloride phases.

The chlorination reaction of M is written by the following equation:

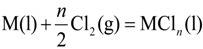
(1)
M refers to alloying element dissolved in liquid aluminum. The equilibrium constant of Equation (1); *K*_1_ is given by:

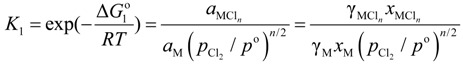
(2)
where 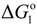, *R*, and *T* are the free energy change of Equation (1), the gas constant, and the absolute temperature; *a*_M_, 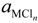, 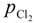 and *P*^0^ are the activity of M, of its chloride, the partial pressure of chlorine gas, and the standard pressure of 1 atm (101,325 Pa/atm); γ_M_, 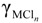 *x*_M_ and 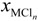 are the activity coefficient of M, of its chloride, and the mole fraction of M in liquid aluminum, of its chloride in salt flux, respectively. Equilibrium constant *K*_1_ can be obtained from the standard free energy change,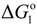 Rearrangement of Equation (2) gives the distribution ratio between salt flux and metal, *L*^salt/metal^:

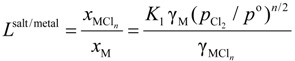
(3)
The activity coefficient of M in molten aluminum, γ_M_, and the activity coefficient of M chloride in salt flux, 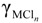, were taken from literatures. The partial pressure of chlorine gas, 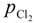, at given temperature and aluminum chloride activity was determined from the following equations and was substituted into Equation (3):

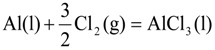
(4)

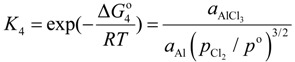
(5)

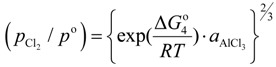
(6)

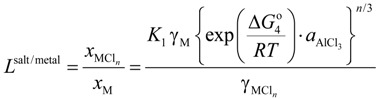
(7)
where, *K*_4_ denotes the equilibrium constant of Equation (4) and 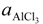 is the activity of aluminum chloride. Equation (6) follows by rearrangement of Equation (5). Consequently, the distribution ratio between salt and metal is expressed as Equation (7). The activity of aluminum was set as unity in the present work due to the condition that the concentration of various elements in Al is low. A larger salt flux-metal distribution ratio *L*^salt/metal^ results in an easier removal of M into the flux phase by chlorination.

The impurities in aluminum can be also removed by oxidation or vaporization. Oxidation and vaporization reactions are considered to obtain oxide slag-metal distribution ratio, *L*^slag/metal^, and gas-metal distribution ratio *L*^gas/metal^ explained in detail in [6,7]. The distribution among Al metal, salt flux, oxide slag and gas phase for various elements were considered in the present work.

For the 43 different elements in liquid aluminum, the activity coefficients were taken from the literature. The standard Gibbs energies of chloride and oxide formation of pure elements were cited from standard thermodynamic tables. All available thermodynamic parameters used in the present work are listed in supporting information (SI). Since no systematic thermodynamic discussions have been done in the past on the behavior of alloying elements by chlorination reaction during the recycling process, our discussion in this paper of the thermodynamic behavior of alloying elements is limited to the Al–M binary alloy. The idea of this paper could be extended to higher alloy systems, if the activities of components in the alloy were available.

## 3. Results and Discussion

The distributions of elements between salt flux and metal phase were evaluated. Adapted temperature was 1073 K which is same of industrial remelting process of aluminum scrap. The initial mole fraction of elements in the metal was fixed as 0.01. The salt flux composition was selected as 45 mol% NaCl–45 mol% KCl–10 mol% AlCl_3_ (Flux A) or 35 mol% NaCl–35 mol% KCl–30 mol% AlCl_3_ (Flux B) because most fluxes used in recycling plant are based on a mixture of NaCl and KCl. The activity of AlCl_3_; at 1073 K was evaluated as 1.1 × 10^−5^ and 3.0 × 10^−5^ in flux A and B by the quasi-chemical model [18], respectively. The activity coefficient of chlorides of Ba, Ca, Ce, Co, Fe, La, Li, Mn, Ni, Sr, and Zn in NaCl–KCl–AlCl_3_ were evaluated by the quasi-chemical model as well [19,20]. The activity coefficient of other elements was assumed to be 1.0. Also, the activity coefficient of MO*_n_* in oxide slag was assumed as unity. The oxygen partial pressure, *P*o_2_, was set at 4.1 × 10^−39^ Pa, which corresponds to the maximum oxygen partial pressure calculated from the equilibrium between Al metal and pure Al_2_O_3_ at 1073 K.

**Figure 1:**
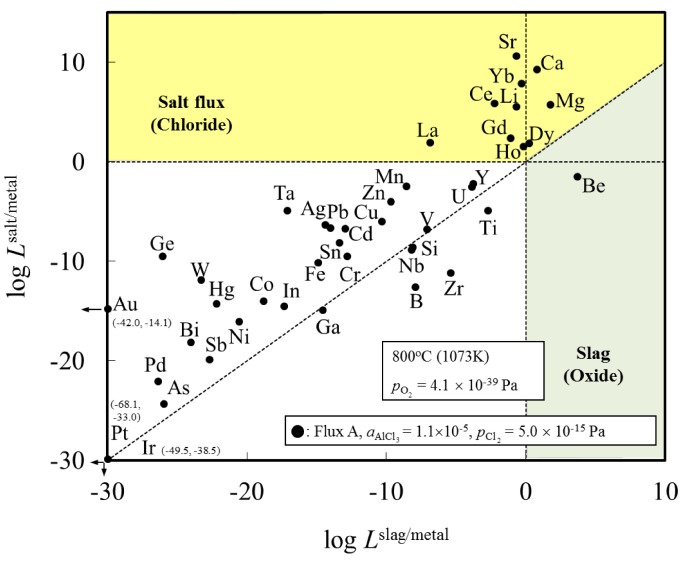
Distribution chart of elements among slag (oxide), salt flux (chloride), and metal phases under the simulated atmosphere of the aluminum melting process.

**Figure 2:**
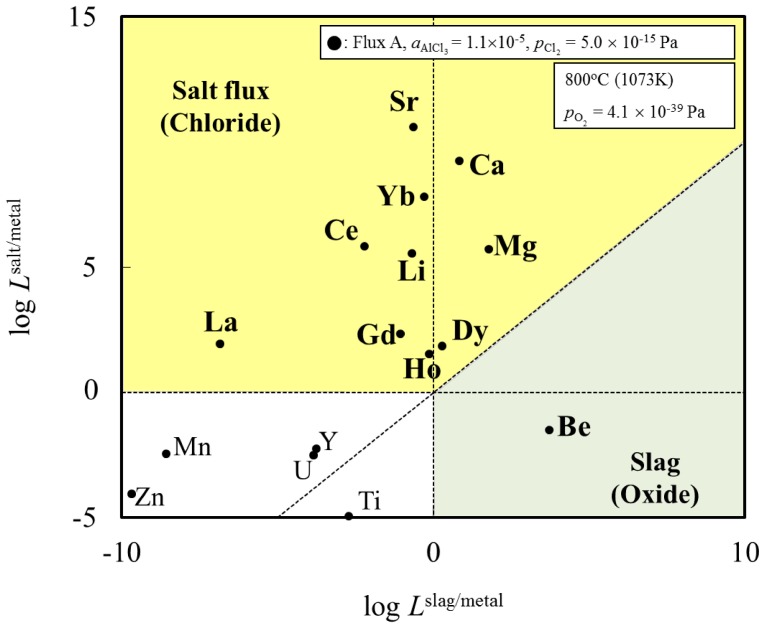
Enlarged section near the origin, taken from Figure 1, showing the distribution of elements among slag (oxide), salt flux (chloride), and metal phases under the simulated atmosphere of the aluminum melting process.

Figure 1 shows the distribution ratio of the elements among the slag (oxide), salt flux (chloride), and metal phases. Elements with a distribution ratio close to unity are impurities which could be removed during aluminum remelting process. Figure 2 shows an enlarged section near the origin of Figure 1. These figures show the results at the temperature of 1073 K when flux A was used. The results for flux B are similar to those for flux A (Table 1).

**Table 1:** Distribution ratio of elements between salt flux (chloride) and metal phases by using flux A or B.

Element	*L* ^salt/metal^		Element	*L* ^salt/metal^	
	Flux A	Flux B		Flux A	Flux B
Ag	−6.4	−6.1	Li	5.5	5.3
As	−24.4	−23.7	Mg	5.7	5.6
Au	−14.1	−13.9	Mn	−2.5	−2.6
B	−12.6	−12.2	Nb	−8.6	−8.2
Be	−1.5	−1.1	Ni	−16.1	−16.2
Bi	−18.1	−17.5	Pb	−6.7	−6.2
Ca	9.24	9.16	Pd	−22.1	−21.6
Cd	−6.8	−6.3	Pt	−33.0	−32.6
Ce	5.8	5.9	Sb	−19.9	−19.2
Co	−14.0	−14.1	Si	−8.9	−8.5
Cr	−8.2	−7.8	Sn	−9.5	−9.1
Cu	−6.1	−5.8	Sr	10.6	10.4
Dy	1.8	2.5	Ta	−4.9	−4.5
Fe	−10.2	−10.2	Ti	−5.0	−4.5
Ga	−14.9	−14.3	U	−2.5	−1.9
Ge	−9.5	−9.1	V	−6.8	−6.4
Gd	2.3	3.0	W	−11.9	−11.5
Hg	−14.3	−13.8	Y	−2.3	−1.6
Ho	1.5	2.1	Yb	7.8	8.2
In	−14.5	−13.9	Zn	−4.1	−4.1
Ir	−38.8	−38.1	Zr	−11.2	−10.8
La	1.9	1.9	–	–	–

Figure 1 indicates that Be can be potentially removed by oxidation (transferred to slag). When either flux A or B is used, Ca, Ce, Dy, Dg, Ho, La, Li, Mg and Sr can be potentially removed by chlorination. The density of SrCl_2_ is higher (2.8 g/cm^3^ at 1073 K) than that of liquid aluminum (2.4 g/cm^3^ at 1073 K), hence, its removal from the remelting furnace requires care, for example, removing after agglomerate formation of SrCl_2_ with other chloride. Other elements, including Cu, Si, Fe, and Mn, are difficult to remove from metal phase, and are likely to be trapped in the metallic aluminum during the remelting process.

**Figure 3:**
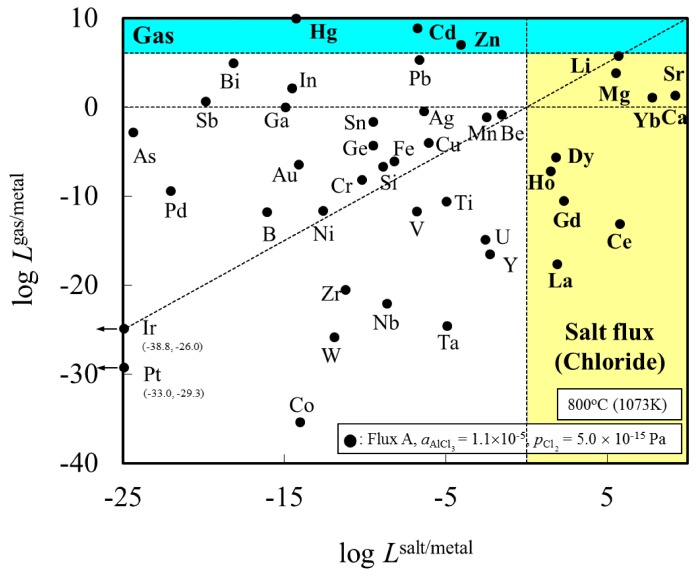
Distribution chart of elements among gas, salt flux (chloride), and metal phases under the simulated atmosphere of the aluminum melting process.

These findings are supported by the work of Mashahadi *et al.*, who experimentally tested the recyclability of aluminum turning by melting them at 1023 K under the presence of salt flux (NaCl–KCl–KF) [14]. It was demonstrated that Cu, Ni, and Si exhibited no essential change in the concentrations after the melting. Their findings support the results of the present work. Shimakage *et al.* [17] measured the distribution of Mg and Zn between KCl–NaCl–AlCl_3_ flux and molten Al at temperatures ranging between 973 and 1073 K. The distribution ratio of Mg and Zn between flux and molten Al was determined from chemical analysis of Mg and Zn content in Al and a mass balance calculation assuming no Mg and Zn evaporation from the sample. They report that Zn and Mg will tend to be distributed in the flux phase. This is not in agreement with our results for Zn in Figure 1 and Figure 2. This difference can be clarified by examining the distribution ratio of elements among Al metal, salt flux and gas phase in Figure 3. Zn, Cd and Hg will evaporate into the gas phase. Hence, it is conjectured that during the experiment of Shimakage *et al.* [17], the Zn content in the Al metal decreased due to its evaporation into the gas phase, rather than being removed with the salt flux.

**Figure 4:**
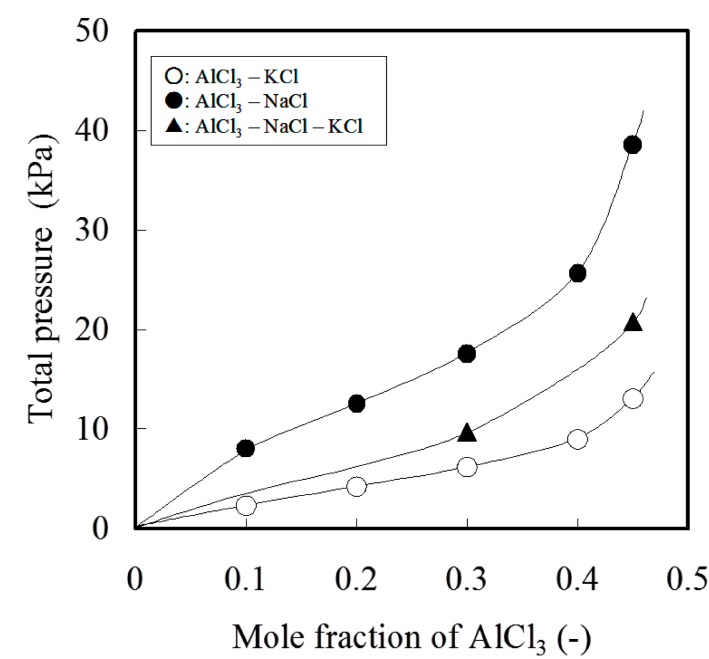
Total pressure of Al chloride equilibrated with AlCl_3_–NaCl, AlCl_3_–KCl and AlCl_3_–NaCl–KCl system at 1073 K.

**Figure 5:**
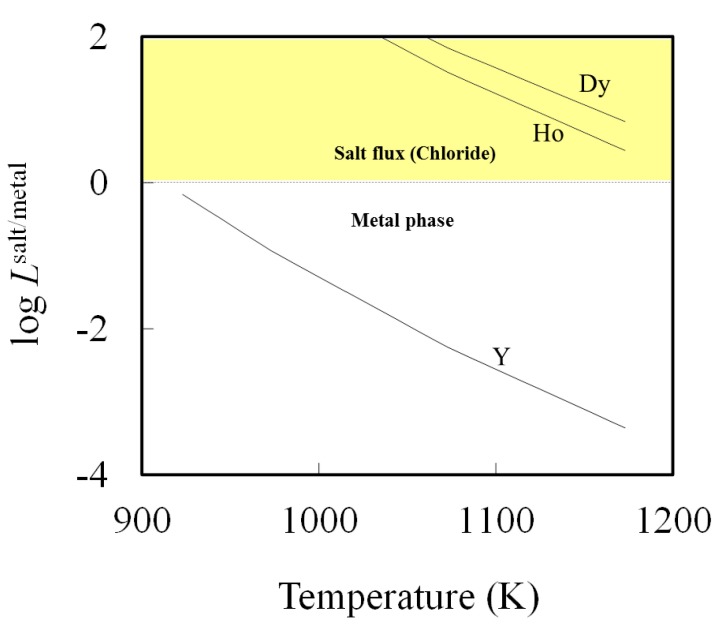
Temperature dependence of element distributions (elements: Dy, Ho and Y) between metal and chloride phase for flux A.

When the flux B was used to treat the aluminum bath, the distribution ratio between salt flux and metal for all elements takes a lower value than when flux A was used. This improvement is due to a higher *P *cl_2_ caused by the larger activity of AlCl_3_ in flux B. This improves the removal capability by increasing the concentration of AlCl_3_ in salt flux. Figure 4 shows a dramatic increase in the total pressure of AlCl_3_ [18]. The significant evaporation loss of aluminum as chloride occurs at higher AlCl_3_ in salt flux. Therefore, the composition of salt flux needs to be maintained to contain less than 40 mol% of AlCl_3_. Figure 5 shows the effect of temperature on the distribution ratio between the salt flux and metal phases for the elements, for which the distribution ratio between salt and molten Al is near unity. Distribution ratios decrease with an increase in the temperature. However, the result indicates the difficulty to controlling the distribution ratio by temperature.

**Figure 6:**
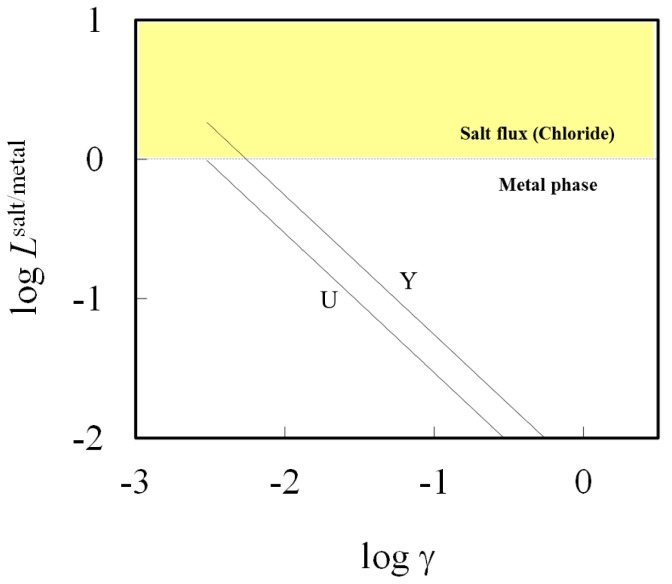
Effect of activity coefficient of chloride on element distributions (elements: U and Y) between metal and chloride phase at 1073 K using flux A.

Figure 6 shows the effect of activity coefficient of chloride product on the distribution ratio, in which Y and U were selected due to poor activity coefficient data for its chloride. The distribution ratio *L*^salt/metal^ increases with a decrease in the activity coefficient of chloride. For instance, if its activity coefficient of YCl_3_ were lower than 0.0061 in flux A at the temperature of 1073 K, Y could be preferentially removed to form YCl_3_ from the aluminum bath. However, a significant decrease in the activity coefficient in salt is unlikely to follow by changing the salt composition due to the limitation of melting temperature and high vapor pressure of aluminum chloride. Hence, it will be difficult to remove most impurities from aluminum and the extent to which it can be improved by controlling the operational parameters during remelting, is limited.

The results in this paper have shown that the chloride flux utilized during aluminum remelting has little effects on impurity removal by chlorination. It was found that salt flux plays a passive role, by protecting the aluminum oxide inclusions formed in aluminum melt. Removal of impurities by oxidation, chlorination and evaporation is difficult under current economic and technological conditions for most impurities and alloying elements in the aluminum melt. In particular, this applies to elements of great importance such as Cu, Si, Fe and Mn. Current recycling practice involves a cascade use of aluminum, from high quality pure aluminum to lower quality alloys, with most aluminum scrap eventually downgraded into die-casting alloys used in automotive engines, which are characterized by high concentrations of alloying elements such as silicon. However, the problem of contaminated aluminum will emerge when the size of the final sink ceases to grow. The difficulty of removing impurities and alloying elements from EoL aluminum implies the importance of avoiding the downgrading its scrap quality whenever possible. Quality management of EoL aluminum, including sorting of aluminum scrap based on product composition, should be promoted to avoid mixing of alloys and contamination by impurities. In contrast to this is the rather remarkable behavior of Mg (a major alloying element) among the salt, oxide, gas and metal phases: the results show that Mg can be removed as chloride than oxide. Typically, when Mg is added as an alloying element, it forms MgO by oxidation and then forms spinel oxide (MgAl_2_O_4_), by the following reactions [21,22,23]:

Mg(l) + 0.5O_2_(g) = MgO(s) (8)

2Al(l) + 1.5O_2_(g) = Al_2_O_3_(s) (9)

MgO(s) + Al_2_O_3_(s) = MgAl_2_O_4_(s) (10)

These reactions indicate the loss of metallic aluminum and the formation of dross with oxidation of Mg, suggesting the possibility of preventing metal loss and dross formation of Al alloy by controlling Mg in remelting processes involving salt flux.

## 4. Conclusions

The equilibrium distribution ratios among the metal, oxide/chloride flux, and gas phases in the aluminum remelting process were investigated by thermodynamic analysis for 43 elements, with the following conclusions:

When either flux 45 mol% NaCl–45 mol% KCl–10 mol% AlCl_3_ or 35 mol% NaCl–35 mol %KCl–30 mol% AlCl_3_ is used, Ca, Ce, Dy, Dg, Ho, La, Li, Mg and Sr can be potentially removed by chlorination. The element Be can be potentially removed by oxidation, while Zn, Cd and Hg will evaporate into the gas phase. Other elements including Cu, Si, Fe, and Mn, are difficult to remove from the metal phase.

Distribution ratios between salt flux and metal, *L*^salt/metal^ decrease with an increase in temperature. However, the extent to which the distribution ratio can be controlled by adjusting the temperature is limited. The distribution ratio *L*^salt/metal^ increases with a decrease in the activity coefficient of chloride. For instance, if the activity coefficient of YCl_3_ is lower than 0.0061 in flux 45 mol% NaCl–45 mol% KCl–10 mol% AlCl_3_ at the temperature of 1073 K, Y can be preferentially removed to form YCl_3_ from the aluminum bath.

## Supplementary Materials

Supplementary File 1
