# Supplementary material for: Thermodynamic Analysis for the Refining Ability of Salt Flux for Aluminum Recycling
Source: Materials (Basel). 2014 Jul 30;7(8):5543–53. doi: 10.3390/ma7085543 (PMC5456205; doi:10.3390/ma7085543)
Supplement: Supplementary File 1 [file materials-07-05543-s001.pdf]

Supporting Information

Calculation of the activity coefficient (γ) and references of interaction parameters.

The activity coefficient γ for element M in an Al–M binary alloy was obtained using Equation (S1) and the thermodynamically assessed Redlich-Kister parameter $\Omega_{\mathrm{Al-M}}$ for the Al–M binary system. Table S1 shows references [S1–S37] for the calculation of γ, Table S2 shows standard Gibbs energy of oxidation and chlorination reaction, and Table S3 shows vapor pressures of metals:

$$RT \ln \gamma_{\mathrm{M}} = {}^{0}\Omega_{\mathrm{Al-M}} x_{\mathrm{Al}}^{2} + {}^{1}\Omega_{\mathrm{Al-M}} x_{\mathrm{Al}}^{2}(4x_{\mathrm{Al}} - 3) + {}^{2}\Omega_{\mathrm{Al-M}} x_{\mathrm{Al}}^{2}(2x_{\mathrm{Al}} - 1)(6x_{\mathrm{Al}} - 5) + {}^{3}\Omega_{\mathrm{Al-M}} x_{\mathrm{Al}}^{2}(2x_{\mathrm{Al}} - 1)^{2}(8x_{\mathrm{Al}} - 7) \quad \text{(S1)}$$

The standard Gibbs energy of oxide formation and the vapor pressure of pure elements were obtained from thermodynamic Tables [S38].

Table S1. References table for estimation of the activity coefficient.

Element	Reference	Element	Reference
Ag	[S1]	Mn	[S3,S20]
As, Ga	[S2,S3]	Na	[S21]
Au	[S4]	Nb	[S22]
B	[S5]	Ni	[S23]
Be	[S6]	Pb	[S3,S24]
Bi	[S7]	Pd	[S25]
Ca	[S3,S8]	Pt	[S26]
Ce	[S9]	Sb	[S27]
Co	[S10]	Sn	[S3,S28]
Cr	[S11]	Sr	[S29]
Cu, Si	[S3,S12]	Ta, V	[S30]
Dy, Gd, Ho	[S13]	Ti	[S31]
Fe, Zr	[S3,S14]	U	[S32]
Ge, Mg	[S3,S15]	Yb	[S33]
In	[S3,S16]	Zn, Y	[S3,S34]
Ir	[S17]	Cd	[S35]
La	[S18]	Hg	[S36]
Li	[S19]	W	[S37]

Table S2. The standard Gibbs energies of chloride formation of pure elements [S38].

Reaction	ΔG^0/J·mol^{-1}	Reaction	ΔG^0/J·mol^{-1}
Ag(l) + 1/2 Cl_2(g) = AgCl(l)	−115990 + 33.1T	Li(l) + 1/2 Cl_2(g) = LiCl(l)	−383552 + 54.0T
Al(l) + 3/2 Cl_2(g) = $AlCl_3$(l)	−668546 + 163.4T	Mg(l) + Cl_2(g) = $MgCl_2$(l)	−594415 + 111.9T
As(s) + 3/2 Cl_2(g) = $AsCl_3$(l)	−302369 + 145.2T	Mn(l) + Cl_2(g) = $MnCl_2$(l)	−445930 + 87.6T
Au(l) + 1/2 Cl_2(g) = AuCl(s)	−48247 + 80.3T	Na(l) + 1/2 Cl_2(g) = NaCl(l)	−426633 + 105.5T
B(s) + Cl_2(g) = BCl_2(g)	−80169 + 42.2T	Nb(l) + Cl_2(g) = $NbCl_2$(s)	−429506 + 141.2T
Be(l) + Cl_2(g) = $BeCl_2$(l)	−479508 + 122.2T	Ni(l) + Cl_2(g) = $NiCl_2$(s)	−318596 + 154.5T
Bi(l) + 3/2 Cl_2(g) = $BiCl_3$(l)	−350039 + 155.3T	Pb(l) + Cl_2(g) = $PbCl_2$(l)	−324163 + 102.9T
Ca(l) + Cl_2(g) = $CaCl_2$(l)	−759317 + 118.9T	Pd(l) + Cl_2(g) = $PdCl_2$(l)	−181232 + 122.2T
Cd(l) + Cl_2(g) = $CdCl_2$(l)	−392786 + 154.6T	Pt(l) + Cl_2(g) = $PtCl_2$(s)	−123623 + 46.5T
Ce(l) + 3/2 Cl_2(g) = $CeCl_3$(l)	−980929 + 173.2T	Sb(l) + 3/2 Cl_2(g) = $SbCl_3$(l)	−420609 + 210.8T
Co(l) + Cl_2(g) = $CoCl_2$(l)	−268739 + 84.2T	Sn(l) + Cl_2(g) = $SnCl_2$(l)	−310534 + 105.4T
Cr(l) + Cl_2(g) = $CrCl_2$(l)	−365290 + 87.8T	Si(l) + Cl_2(g) = $SiCl_2$(g)	−220359 − 5.9T
Cu(l) + 1/2 Cl_2(g) = CuCl(l)	−146215 + 30.8T	Sr(l) + Cl_2(g) = $SrCl_2$(l)	−799640 + 129.7T
Dy(l) + 3/2 Cl_2(g) = $DyCl_3$(l)	−959721 + 195.1T	Ta(l) + Cl_2(g) = $TaCl_3$(s)	−572391 + 216.7T
Fe(l) + Cl_2(g) = $FeCl_2$(l)	−302026 + 76.2T	Ti(l) + Cl_2(g) = $TiCl_2$(s)	−52510 + 162.7T
Ga(l) + 3/2 Cl_2(g) = $GaCl_3$(l)	−513269 + 210.2T	U(l) + 3/2 Cl_2(g) = UCl_2(l)	−812875 + 169.4T
Gd(l) + 3/2 Cl_2(g) = $GdCl_3$(l)	−960475 + 188.2T	V(l) + Cl_2(g) = VCl_2(s)	−467862 + 152.2T
Ge(l) + Cl_2(g) = $GeCl_2$(l)	−210245 − 7.3T	W(l) + Cl_2(g) = WCl_2(s)	−288374 + 122.4T
Hg(l) + Cl_2(g) = $HgCl_2$(l)	−206519 + 109.5T	Y(l) + 3/2 Cl_2(g) = YCl_3(l)	−960194 + 188.0T
Ho(l) + 3/2 Cl_2(g) = $HoCl_3$(l)	−965130 + 200.3T	Yb(l) + Cl_2(g) = $YbCl_2$(s)	−801829 + 144.5T
In(l) + 3/2 Cl_2(g) = $InCl_3$(s)	−531996 + 240.7T	Zn(l) + Cl_2(g) = $ZnCl_2$(l)	−411693 + 142.4T
Ir(s) + 3/2 Cl_2(g) = $IrCl_3$(s)	−268113 + 255.4T	Zr(l) + Cl_2(g) = $ZrCl_2$(l)	−412629 + 114.8T
La(l) + 3/2 Cl_2(g) = $LaCl_3$(l)	−994064 + 170.9T	–	

References

S1. Witusiewicz, V.T.; Hecht, U.; Fries, S.G.; Rex, S. The Ag–Al–Cu system: Part I: Reassessment of the constituent binaries on the basis of new experimental data. *J. Alloys Compd.* **2004**, *385*, 133–143.

S2. Li, C.; Li, J.-B.; Du, Z.; Lu, L.; Zhang, W. A thermodynamic reassessment of the Al–As–Ga System. *J. Phase Equilib.* **2001**, *22*, 26–33.

S3. SGTE Solution Database v2. Available online: http://www.sgte.org/ (accessed on 29 July 2014).

S4. Li, M.; Li, C.; Wang, F.; Luoa, D.; Zhang, W. Thermodynamic assessment of the Al–Au system. *J. Alloys Compd.* **2004**, *385*, 199–206.

S5. Mirković, D.; Gröbner, J.; Schmid-Fetzer, R.; Fabrichnaya, O.; Lukas, H.L. Experimental study and thermodynamic re-assessment of the Al–B system. *J. Alloys Compd.* **2004**, *384*, 168–174.

S6. Pan, Z.; Du, Y.; Huang, B.Y.; Liu, Y.; Wang, R.C. A thermodynamic description of the Al–Be system: Modeling and experiment. *Calphad* **2004**, *28*, 371–378.

S7. Gröbner, J.; Mirković, D.; Schmid-Fetzer, R. Monotectic four-phase reaction in Al–Bi–Zn alloys. *Acta Mater.* **2005**, *53*, 3271–3280.

S8. Ozturk, K.; Chen, L.-Q.; Liu, Z.-K. Thermodynamic assessment of the Al–Ca binary system using random solution and associate models. *J. Alloys Compd.* **2002**, *340*, 199–206.
S9. Bo, H.; Jin, S.; Zhang, L.G.; Chen, X.M.; Chen, H.M.; Liu, F.; Zheng, L.B.; Jin, Z.P. Thermodynamic assessment of Al–Ce–Cu system. *J. Alloys Compd.* **2009**, *484*, 286–295.
S10. Ohtani, H.; Yamano, M.; Hasebe, M. Thermodynamic analysis of the Co–Al–C and Ni–Al–C systems by incorporating ab initio energetic calculations into the CALPHAD approach. *Calphad* **2004**, *28*, 177–190.
S11. Liang, Y.; Guo, C.; Li, C.; Du, Z. Thermodynamic modeling of the Al–Cr system. *J. Alloys Compd.* **2008**, *460*, 314–319.
S12. He, C.-Y.; Du, Y.; Chen, H.-L.; Xu, H. Experimental investigation and thermodynamic modeling of the Al-Cu-Si system. *Calphad* **2009**, *33*, 200–210.
S13. Cacciamani, G.; De Negri, S.; Saccone, A.; Ferro, R. The Al–R–Mg (R = Gd, Dy, Ho) systems. Part II: Thermodynamic modelling of the binary and ternary systems. *Intermetallics* **2003**, *11*, 1135–1151.
S14. Guo, C.; Du, Z.; Li, C.; Zhang, B.; Tao, M. Thermodynamic description of the Al–Fe–Zr system. *Calphad* **2008**, *32*, 637–649.
S15. Islam, F.; Thykadavil, A.K.; Medraj, M. A computational thermodynamic model of the Mg–Al–Ge system. *J. Alloys Compd.* **2006**, *425*, 129–139.
S16. Kim, S.S.; Sanders, T.H., Jr. Thermodynamic assessment of the metastable liquidid in the Al–In, Al–Bi and Al–Pb systems. *Modelling Simul. Mater. Sci. Eng.* **2006**, *14*, 1181–1188.
S17. Abe, T.; Kocer, C.; Ode, M.; Murakami, H.; Yamabe-Mitarai, Y.; Hashimoto, K.; Onodera, H. Thermodynamic re-assessment of the Al–Ir system. *Calphad* **2008**, *32*, 686–692.
S18. Yin, F.; Su, X.; Li, Z.; Huang, M.; Shi, Y. A thermodynamic assessment of the La–Al system. *J. Alloys Compd.* **2000**, *302*, 169–172.
S19. Hallstedt, B.; Kim, O. Thermodynamic assessment of the Al–Li system. *Int. J. Mater. Res.* **2007**, *98*, 961–969.
S20. Liu, X.; Hao, S. A thermodynamic calculation of the Fe–Mn–Al ternary system. *Calphad* **1993**, *17*, 79–91.
S21. Qiu, C.; Opalka, S.M.; Løvvik, O.M.; Olson, G.B. Thermodynamic modeling of the Na–Al–Ti–H system and Ti dissolution in sodium alanates. *Calphad* **2008**, *32*, 624–636.
S22. Zhu, Z.; Du, Y.; Zhang, L.; Chen, H.; Xu, H.; Tang, C. Experimental identification of the degenerated equilibrium and thermodynamic modeling in the Al–Nb system. *J. Alloys Compd.* **2008**, *460*, 632–638.
S23. Ansara, I.; Dupin, N.; Lukas, H.L.; Sundman, B. Thermodynamic assessment of the Al–Ni system. *J. Alloys Compd.* **1997**, *247*, 20–30.
S24. Liu, Y.; Liang, D. A contribution to the Al–Pb–Zn ternary system. *J. Alloys Compd.* **2005**, *403*, 110–117.
S25. Li, M.; Li, C.; Wang, F.; Zhang, W. Thermodynamic assessment of the Al–Pd system. *Intermetallics* **2006**, *14*, 39–46.
S26. Lu, X.-G.; Sundman, B.; Ågren, J. Thermodynamic assessments of the Ni–Pt and Al–Ni–Pt systems. *Calphad* **2009**, *33*, 450–456.

S27. Balakumar, T.; Medraj, M. Thermodynamic modeling of the Mg–Al–Sb system. *Calphad* **2005**, *29*, 24–36.
S28. Ansara, I.; Bros, J.P.; Gambino, M. Thermodynamic analysis of the germanium-based ternary systems (Al–Ga–Ge, Al–Ge–Sn, Ga–Ge–Sn). *Calphad* **1979**, *3*, 225–233.
S29. Wang, C.; Jin, Z.; Du, Y. Thermodynamic modeling of the Al–Sr system. *J. Alloys Compd.* **2003**, *358*, 288–293.
S30. Du, Y.; Wenzel, R.; Schmid-Fetzer, R. Thermodynamic analysis of reactions in the Al–N–Ta and Al–N–V systems. *Calphad* **1989**, *22*, 43–58.
S31. Witusiewicz, V.T.; Bondar, A.A.; Hecht, U.; Rex, S.; Velikanov, T.Y. The Al–B–Nb–Ti system: III. Thermodynamic re-evaluation of the constituent binary system Al–Ti. *J. Alloys Compd.* **2008**, *465*, 64–77.
S32. Wang, J.; Liu, X.J.; Wang, C.P. Thermodynamic modeling of the Al–U and Co–U systems. *J. Nucl. Mater.* **2008**, *374*, 79–86.
S33. Meng, F.G.; Zhang, L.G.; Liu, H.S.; Liu, L.B.; Jin, Z.P. Thermodynamic optimization of the Al–Yb binary system. *J. Alloys Compd.* **2008**, *452*, 279–282.
S34. Liu, X.J.; Wen, M.Z.; Wang, C.P.; Pan, F.S. Thermodynamic assessment of the Zn–Y and Al–Zn–Y systems. *J. Alloys Compd.* **2008**, *452*, 283–290.
S35. K. Oikawa. Private communication, 2009.
S36. Al–Hg (Data from SGTE Alloy Database). Available online: http://www.crct.polymtl.ca/fact/phase_diagram.php?file=Al-Hg.jpg&dir=SGTE (accessed on 29 July 2014).
S37. Kaufman, L. *PKP—Kaufman Binary Alloy Database, ver. 1.1*; Man Labs Inc.: New York, NY, USA, 2003.
S38. Thermochemical Data of Pure Substances. Available online: http://zennet.kr/scan/Thermochemical%20Data%20of%20Pure%20Substances%20PART%20I%28ocr%29.pdf (accessed on 29 July 2014).
